# Supplementary material for: Enhancing the Capacity and Stability by CoFe2O4 Modified g-C3N4 Composite for Lithium-Oxygen Batteries
Source: Nanomaterials (Basel). 2021 Apr 22;11(5):1088. doi: 10.3390/nano11051088 (PMC8146125; doi:10.3390/nano11051088)
Supplement: Supplementary file 1 [file nanomaterials-11-01088-s001.zip › nanomaterials-1168094-supplementary.pdf]

nanomatrials

Surporting Information

# Enhancing the Capacity and Stability by $CoFe_2O_4$ Modified $g\text{-}C_3N_4$ Composite for Lithium-Oxygen Batteries

**Xiaoya Li [1], Yajun Zhao [1], Lei Ding [1], Deqiang Wang [1], Qi Guo [1], Zhiwei Li [1], Hao Luo [1,*], Dawei Zhang [1] and Yan Yu [2,*]**

[1] School of Chemistry and Chemical Engineering, Hefei University of Technology, Hefei 230009, China; Sabrina_lxy@163.com (X.L.); zyj251264@163.com (Y.Z.); dlei1107@163.com (L.D.); wondreful26@163.com (D.W.); 18297919084@163.com (Q.G.); Zhiwei.Li@hfut.edu.cn (Z.L.); zhangdw@ustc.edu.cn (D.Z.)

[2] Department of Materials of Science and Engineering, University of Science and Technology, Heifei 230026, China

* Correspondence: luohao@hfut.edu.cn (H.L.); yanyumse@ustc.edu.cn (Y.Y.)

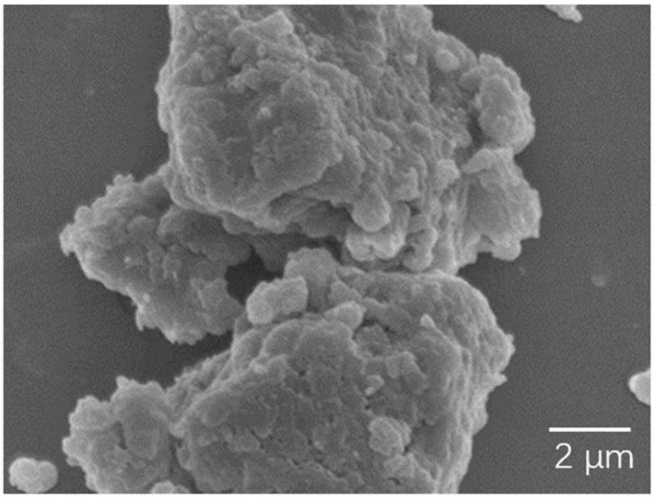


**Figure S1.** SEM image of bulk g-$C_3N_4$.

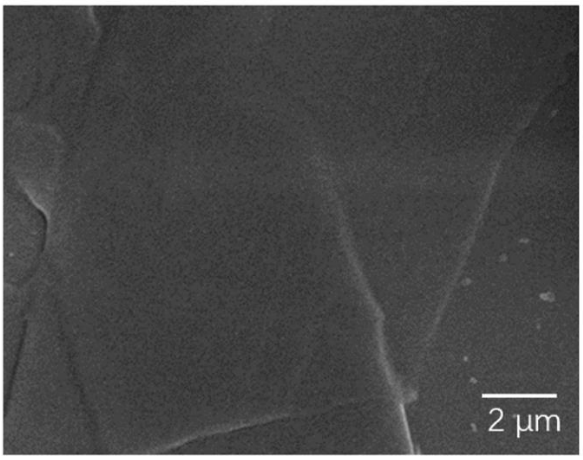


**Figure S2.** SEM image of flake g-$C_3N_4$.

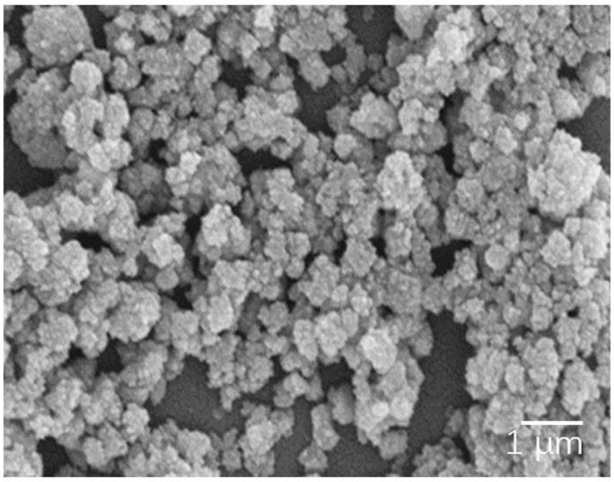


**Figure S3.** SEM image of $CoFe_2O_4$ cathode.

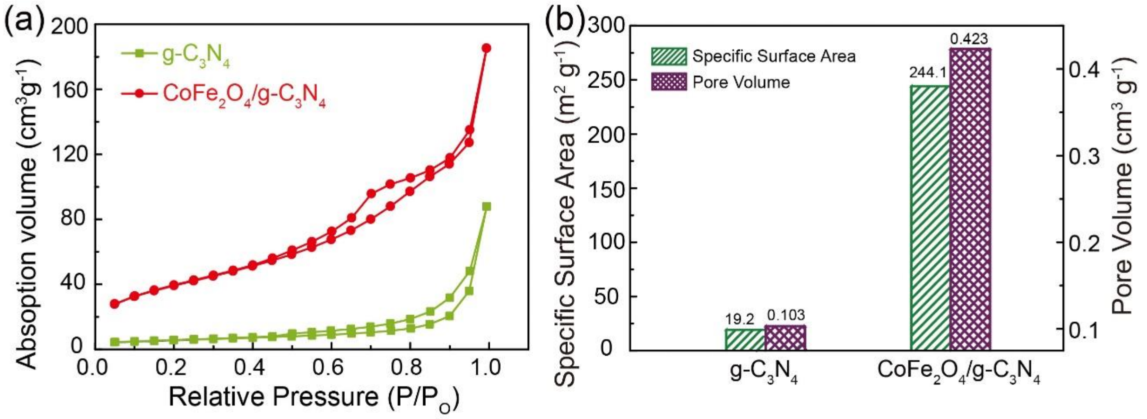


**Figure S4.** (**a**) $N_2$ adsorption-desorption isotherms and (**b**) specific surface area and pore volume of g-$C_3N_4$ and $CoFe_2O_4$/g-$C_3N_4$.
